# Engineering live cell surfaces with polyphenol-functionalized nanoarchitectures

**DOI:** 10.1039/d4sc07198k

**Published:** 2025-02-11

**Authors:** Yunxiang He, Qinling Liu, Yuanmeng He, Siqi Deng, Junling Guo

**Affiliations:** a BMI Center for Biomass Materials and Nanointerfaces, National Engineering Laboratory for Clean Technology of Leather Manufacture, Ministry of Education Key Laboratory of Leather Chemistry and Engineering, College of Biomass Science and Engineering, Sichuan University Chengdu Sichuan 610065 China junling.guo@scu.edu.cn junling.guo@ubc.ca; b Tea Refining and Innovation Key Laboratory of Sichuan Province, College of Horticulture, Sichuan Agricultural University Chengdu Sichuan 611130 China; c Bioproducts Institute, Department of Chemical and Biological Engineering, The University of British Columbia Vancouver BC V6T 1Z4 Canada; d State Key Laboratory of Polymer Materials Engineering, Sichuan University Chengdu Sichuan 610065 China

## Abstract

Cell surface functionalization has emerged as a powerful strategy for modulating cellular behavior and expanding cellular capabilities beyond their intrinsic biological limits. Natural phenolic molecules present as ‘green’ and versatile building blocks for constructing cell-based biomanufacturing and biotherapeutic platforms. Due to the abundant catechol or galloyl groups, phenolic molecules can dynamically and reversibly bind to versatile substrates *via* multiple molecular interactions. A range of self-assembled cytoadhesive polyphenol-functionalized nanoarchitectures (cytoPNAs) can be formed *via* metal coordination or macromolecular self-assembly that can rapidly attach to cell surfaces in a cell-agnostic manner. Additionally, the cytoPNAs attached on the cell surface can also provide active sites for the conjunction of bioactive payloads, further expanding the structural repertoire and properties of engineered cells. This Perspective introduces the wide potential of cytoPNA-mediated cell engineering in three key applications: (1) creating inorganic–organic biohybrids as cell factories for efficient production of high-value chemicals, (2) constructing engineered cells for cell-based therapies with enhanced targeting specificity and nano–bio interactions, and (3) encapsulating microbes as biotherapeutics for the treatment of gastrointestinal tract-related diseases. Collectively, the rapid, versatile, and modular nature of cytoPNAs presents a promising platform for next-generation cell engineering and beyond.

## Abstract 2

Polyphenol-functionalized nanoarchitectures (cytoPNAs) provide a modular and cell independent strategy to create biohybrids for cell engineering, with improved production for biomanufacturing and enhanced efficacy for cell-based therapies.

## Graphical abstract

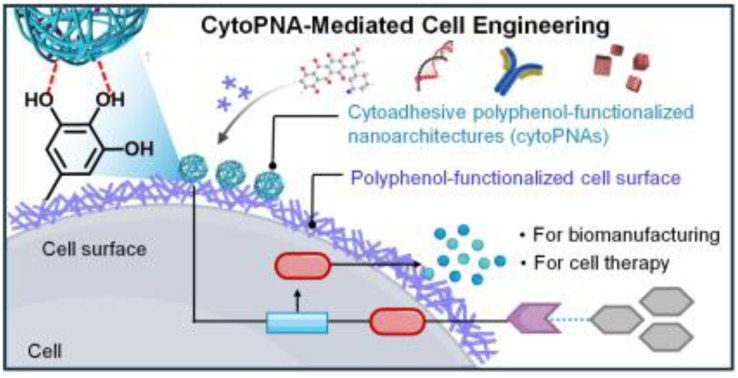

## 1 Introduction

Synthetic biology and cell surface functionalization are two prevalent strategies of cell engineering that have significantly enhanced our understanding on the underlying mechanisms governing various biological behaviors in cells, enabling control over cellular interactions and new functionalities that go beyond natural biological limits.^1,2^ Cell surface functionalization emerges as a rapid and versatile tool to integrate material systems (such as inorganic particles, organic compounds, and biological macromolecules) with living cells. For example, the introduction of cyto-attached nanosystems can shield the cells from external environmental stresses such as toxins, immune responses, or harsh conditions, while maintaining their viability and functionality. Moreover, cell surface functionalization also allows precise control over cell–environment interactions, introduces new functionalities, and enhances biocompatibility, thereby broadening the potential applications of cells in biomanufacturing and cell-based therapies.^3–5^

**Fig. 1:**
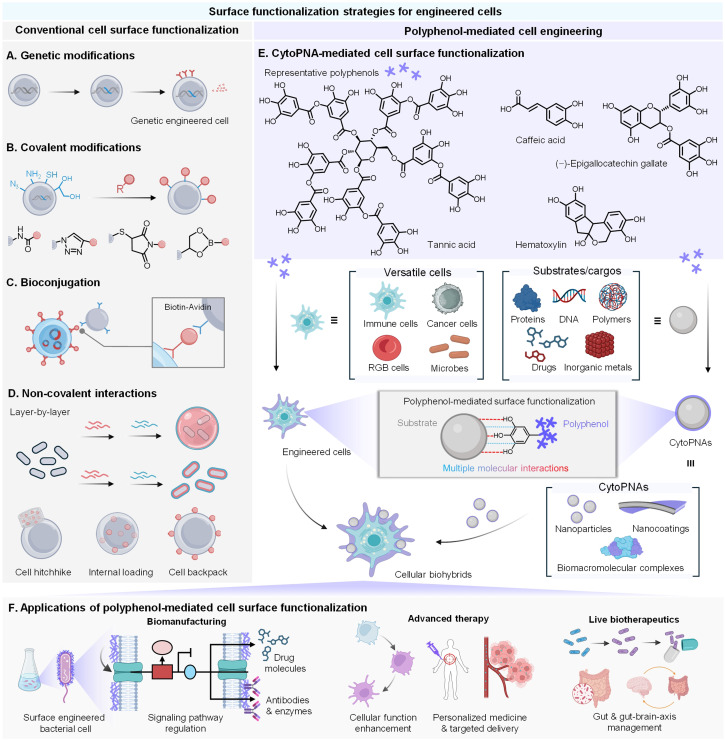
Strategies for cell surface functionalization to construct engineered cells with enhanced properties and functions. (A) Genetic strategies for cell surface functionalization. (B) Chemical covalent functionalization strategies. (C) Bioconjugation-based strategies *via* biotin–avidin interactions for cell surface functionalization. (D) Non-covalent strategies for efficient multiscale cell wrapping and varied patterns of cargo loading. (E) Polyphenol-based strategies for cytoPNA preparation exhibit a modular and convenient approach to providing functional cellular biohybrids based on microbes and cells. (F) The applications of cytoPNA-mediated cell surface functionalization for cellular biohybrids to facilitate the development of biomanufacturing, advanced therapy, and live biotherapeutics (created with https://www.BioRender.com).

Several approaches have been developed for cell surface functionalization, ranging from genetic methods and chemical synthesis techniques to non-covalent interactions (Fig. 1A–D).^6,7^ Genetic methods involve engineering cells to express specific surface proteins, enabling the attachment of functional groups or the self-assembly of protective layers.^8^ Chemical synthesis techniques achieve stable covalent attachments between the reactive functional groups to the synthetic molecules or polymers on the cell surface.^9^ While effective, these approaches face potential concerns regarding intricate and expensive genetic modifications, limited flexibility and adaptability of functionalization, and compromises in long-term cell viability. In contrast, non-covalent methods, especially those based on supramolecular interactions, offer significant advantages due to their reversible, biocompatible, and modular nature.^10^ These methods use weak interactions like hydrogen bonding and π–π stacking to modify the cell surface, providing a non-invasive and adaptable platform for adding functional motifs dynamically.^11^ More importantly, the single-cell encapsulation can be tailored for multifunctionality while maintaining cell integrity.

Natural polyphenols, containing rich catechol or galloyl groups in their molecular structures, exhibit capabilities to bind with various substrates *via* multiple molecular interactions.^12–14^ These unique structural and chemical features of polyphenols facilitate the formation of a range of polyphenol-functionalized nanoarchitectures (PNAs), including nanocomplexes,^15^ nanoparticles,^16,17^ and nanocoatings,^18,19^ driven by metal coordination or macromolecular self-assembly. Importantly, the catechol or galloyl groups on the surface of PNAs also endow the functional substrates with cytoadhesion capability to attach to a different type of cells (referred to as cytoadhesive PNAs, cytoPNAs), offering a cell-independent, modular cell surface functionalization.^20^ Our group, along with others, demonstrated that cytoPNAs can be used to construct cell-based biohybrids integrated with various functional cargoes such as inorganic nanoparticles, therapeutic agents, enzymes, or imaging probes for the applications of biomanufacturing and cell-based therapies (Fig. 1E and F).^21–23^ The dynamic and modular nature makes cytoPNAs highly advantageous for applications requiring adaptable and reversible modifications, positioning them as a promising strategy for next-generation cell engineering.

This Perspective aims to introduce the rapidly developing research and applications of cytoPNAs in cell surface functionalization, showcasing its transformative potential in cell-based biomanufacturing and biotherapeutics. The modular and rational design of cytoPNAs opens new avenues for enhancing cellular functions, creating biohybrids with tailored properties, and ultimately, revolutionizing the next-generation biosynthetic production and personalized medicine.

## 2 Molecular interactions in cytoPNAs and engineered cells

Polyphenol-based materials can directly interact with cells and functional cargoes to construct engineered cells and cytoPNAs, respectively, through covalent and diverse non-covalent binding mechanisms, serving as a platform for constructing functional biointerfaces.^24^ Understanding these interactions is crucial for optimizing the functionality and stability of cytoPNAs and derived cellular biohybrids.

### 2.1 Covalent interactions

**Fig. 2:**
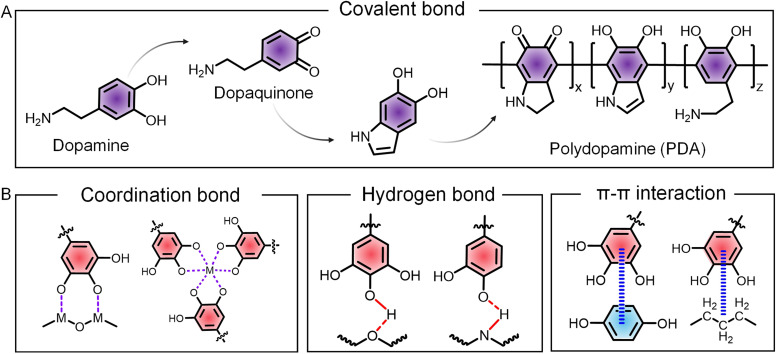
Schematic of polyphenol-mediated multiple molecular interactions for the formation of cytoPNAs. (A) Representative covalent interactions induced by PDA and derivatives. (B) Chemical illustration on the characteristic non-covalent interactions mediated by phenolic groups in polyphenols and cytoPNAs.

Engineered cells with dopamine and its derivatives are pioneer examples of covalent decoration of phenolic molecules to form nanocoatings on cells.^25^ The catecholamine of dopamine undergoes spontaneous oxidation in alkaline conditions to form dopaquinone, followed by further oxidation and polymerization to yield a complex network of polydopamine (PDA) containing covalently linked phenolic units attached on the surface of both cells and functional cargoes, enabling the construction of engineered cells and cytoPNAs (Fig. 2A). In addition, the PDA-induced cytoPNAs form a strong adhesive layer on cell surfaces, providing a stable interface for further functionalization. The catechol groups in PDA can react with nucleophilic groups such as amines and mercaptans present on membrane proteins, forming covalent bonds and enhancing the stability and longevity of the engineered cell surface. For instance, therapeutic agents, imaging probes, or other bioactive molecules can be covalently linked to the PDA coating through reactions such as Michael addition or Schiff base reactions. PDA-induced cytoPNAs can assist the precise delivery of these cargoes to specific cellular targets,^26^ enhance the efficacy and specificity of cell-based therapies,^27^ and also expand potential applications in catalysis and sensing.^28^

### 2.2 Non-covalent interactions

Non-covalent interactions, such as coordination, hydrogen bonding, hydrophobic forces, and π–π stacking, are the predominant methods for the cytoPNAs and engineered cells (Fig. 2B).^29^ Coordination with metal ions is a characteristic reaction of polyphenols, where the deprotonated phenolic groups donate the electrons to metal ion centers to form the coordination bonds. The coordination bond dominates the structure and properties of cytoPNAs consisting of polyphenols and metal ions. Hydrogen bonding is formed between the phenolic groups in polyphenols with various hydroxyl and carboxyl groups on cell or functional cargo surfaces, which is characterized by bridging the oxygen atom of the phenolic group and a highly electronegative atom (such as oxygen or nitrogen) on the cell or cargo surface *via* a hydrogen atom. Hydrophobic interactions and π–π interactions are significant in forming cytoPNAs and are raised by the hydrophobic aromatic rings. The tendency of hydrophobic groups to aggregate in an aqueous environment and the overlap of π-electrons significantly enhance the attachment of phenolic molecules on the cell and cargo surface.

Typically, tannic acid (TA) and ferric ions (Fe^3+^) can form a stable metal–phenolic network on a variety of substrate surfaces from metal oxide rods, polymeric nanowires, and nanosheets to biological cells.^30^ These substrates with different sizes, shapes, and compositions are transformed into cytoPNAs by the coordination-induced surface functionalization and are further constructed into complex superstructures through metal ion-mediated intermolecular interactions and particle interlocking. Particularly, the non-covalent strategies are effective for cellular biohybrids with cytoPNAs and impart stimuli responsiveness such as pH or temperature by utilizing the dynamic properties of the coordination bonds.^12,31^ These phenolic modifications can be tailored for functional applications, such as the immobilization of growth factors like vascular endothelial growth factor to enhance endothelial cell survival and tissue regeneration.

Non-covalent interactions offer reversibility and adaptability, which are generally considered less invasive and promising preservation for cell viability and functions. On the other hand, the dynamic nature of non-covalent interactions suggests a less stable form compared with that of covalent bonding, especially in the high temperatures of some biomanufacturing processes or the complicated and fluctuated physical conditions. Attention must be paid to designing cytoPNAs, ensuring that the interactions are strong enough to maintain functionality while still allowing for reversibility and adaptability. Hence, covalent modifications are still prevalently employed in many practices due to their high stability and permanence, which are essential for long-term applications and in environments where reversibility is not required.

Collectively, the dynamic and tunable nature of phenolic-based materials provides a versatile toolkit for engineering cell–material interactions. The integration of phenolic materials onto cell and functional cargo surfaces enhances cell survival, modulates cellular behavior, and introduces new functionalities. The ability to construct cytoPNAs and biohybrids with polyphenols opens the door to a wide range of applications, from biomanufacturing to personalized medicine and cell-based therapies.

## 3 Inorganic–organic biohybrids for biosynthesis

Cell engineering aims to effectively regulate and control cell behaviors to augment the properties or achieve new functions, where engineered cells as ‘cell factories’ for designed chemical production stand as a representative practice. The underlying mechanism is to improve the efficiency of energy utilization.

**Fig. 3:**
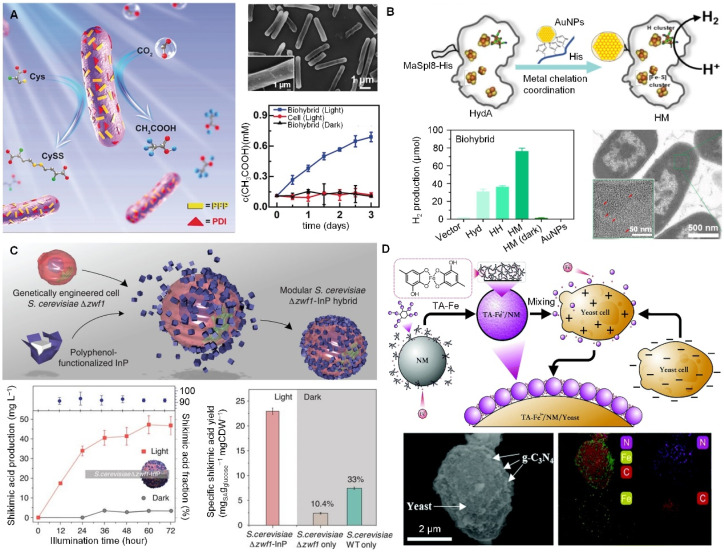
Cell surface functionalization aimed at regulating cellular pathways for the production of high-value chemicals. (A) Organic semiconductor–bacteria biohybrid system for the production of acetic acid, where the semiconductor was constructed on the bacterial surface, affording higher hole/electron separation efficiency and ensuring the efficient electron transfer to bacteria for promoting biosynthesis.^34^ Copyright 2020, Wiley-VCH GmbH. (B) Assembly of gold nanoparticles (AuNPs) with hydrogenases in membrane-free organelles and a comparison of hydrogen production in different systems.^35^ Copyright 2024, Elsevier. (C) Assembly of *Saccharomyces cerevisiae*–InP biohybrids and their effect on the production of shikimic acids.^36^ Copyright 2018, AAAS. (D) TA-modified yeast biohybrids regulate the balanced levels of NADH and NAD within the yeast.^37^ Copyright 2021, Royal Society of Chemistry.

Photosynthesis is the most efficient natural process for using light and covert into chemical energy, driving the chemical conversion of CO_2_ into organic matter and splitting water to produce oxygen.^32^ To artificially harness this potential, light-harvesting semiconductors and metabolically engineered microbes are paired to enable the biohybrids to efficiently carry out the complex biosynthetic pathways for aimed chemicals. The modified anaerobic bacterium *Moorella thermoacetica* can absorb cadmium ion (Cd^2+^) from the solution by the surface transport protein, together with the dispersed cysteine, producing the insoluble cadmium sulfide (CdS) nanoparticles deposited on the bacterial surface. CdS can absorb light and pass the excited photoelectrons to the bacteria to produce acetic acid from CO_2_.^33^ The production of acetic acid can be optimized with high selectivity and yields by turning the composition of inorganic–organic biohybrids. Organic semiconductor–bacteria biohybrid systems have also been reported to efficiently increase the production of acetic acid. Perylene diimide derivatives with cationic electron-transporting (n-type) and hydrophobic hole-transporting (p-type) structures were constructed to form p–n heterojunction and coated to the bacterial surface, affording higher hole/electron separation efficiency and ensuring the efficient electron transfer to bacteria for promoting the biosynthesis (Fig. 3A).^34^ A more complicated system has been established by integrating Au nanoparticle enzymes into membrane-free organelles in non-photosynthetic bacteria, which greatly improves solar energy conversion and hydrogen production (Fig. 3B).^35^ All these biohybrids demonstrate the regulatory capabilities of functional cargoes to cellular behavior when integrated as a biohybrid *via* cell surface functionalization.

The cytoPNAs have been applied to a series of microbial cells to construct cellular biohybrids for producing high-value chemicals from simple inputs as a cell factory. A representative example would be the cytoPNAs composed of TA-functionalized indium phosphide (InP) nanoparticles to construct cellular biohybrids for the carbon- and energy-efficient production of shikimic acid (Fig. 3C).^36^ The TA-InP cytoPNAs adhered to the surface of the engineered yeast *Saccharomyces cerevisiae* to facilitate the transfer of the electrons from InP to the NADP^+^ to produce NADPH, supporting the continuous biosynthesis of shikimic acid. Similar results have been observed with the biohybrids composed of bismuth (Bi)- and graphitic carbon nitride (g-C_3_N_4_)-containing cytoPNAs on yeast cells, where the NADH (important intermediate in the electron pathway in cellular energy conversion and metabolism) content is found to increase significantly (Fig. 3D).^37,38^

Conventional strategies in biomanufacturing often rely solely on genetically engineered biological systems. For example, genetically engineered microbes are generally highly efficient in terms of selectivity and specificity but can be limited by their metabolic capacity and sensitivity to environmental conditions.^5,10^ Inorganic–organic biohybrids combine the strengths of both systems and can enhance overall efficiency and versatility. The combination generally produces synergistic effects that result in higher selectivity and productivity compared to using the biological system alone.^36,37^ However, there are certain limitations to consider. The fabrication and maintenance of these hybrid systems can be complex and costly, and the stability and reproducibility of the hybrid interface under various conditions remain a challenge. Additionally, the scalability of these systems for industrial applications may require further optimization. The integration of inorganic material-based cytoPNAs with biological systems demonstrates the remarkable potential for the efficient production of high-value chemicals and the conversion of solar energy into chemical energy.

## 4 Engineered cells for advanced cell-based therapy

Adoptive cell therapy (ACT) is an emerging and highly promising approach in cancer immunotherapy, where immune cells (typically T cells) are engineered to target and eliminate diseased cells. Conventionally, the T cells are genetically engineered by introducing transgenes, such as chimeric antigen receptors (CARs) or tumor-specific T cell receptors (TCRs), to enhance their ability to recognize and attack cancer cells. Significant successes have been achieved with the FDA-approved tisagenlecleucel and axicabtagene ciloleucel, which are two products that have revolutionized the treatment of hematologic malignancies.^39,40^ Material-based engineering involves the use of biomaterials to modify or enhance the immune cell functions *via* surface modification, encapsulation, and the delivery of bioactive agents and provides a less invasive and dynamic strategy for realizing the survival increase, targeting specificity, and rapid activation of immune cells.^41^ Diverse functional motifs such as biorthogonal chemical groups,^42^ synthetic polymers,^43^ and biomacromolecules^44^ have been employed to modify cell surface *via* electrostatic interactions, hydrophobic insertion, and bioconjugation reactions, holding potential in therapies.

**Fig. 4:**
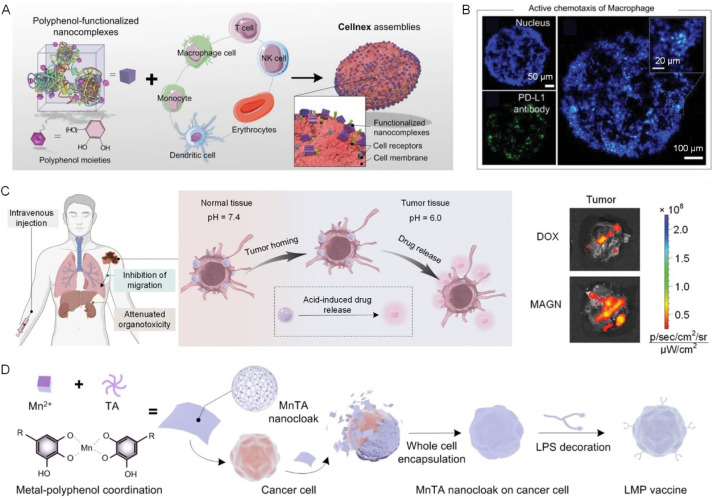
Engineered cells *via* polyphenol-based or cytoPNA-based cell surface functionalization for advanced cell-based therapy. (A) Modular assembly of ‘Cellnex’ by assembling polyphenol-functionalized bioactive nanocomplexes on cells. (B) Therapeutic antibodies (green) can be delivered precisely by the macrophages to the tumor spheroid and nucleus (blue).^45^ Copyright 2020, Wiley-VCH GmbH. (C) Schematic illustration on the macrophage-based biohybrids to load chemotherapeutic drugs with responsive release properties for antitumor applications. The biohybrid system demonstrated improved targeted delivery efficiency.^46^ Copyright 2023, Wiley-VCH GmbH. (D) A schematic illustration of the preparation of a novel whole-cell cancer vaccine (referred to as LMP vaccine) developed by cloaking living cancer cells with lipopolysaccharide (LPS)-modified manganese-phenolic networks (MnTA nanocloaks).^49^ Copyright 2023, Wiley-VCH GmbH.

Due to the strong binding capabilities of catechol and galloyl groups, polyphenols exhibit unique advantages in cell surface functionalization and the creation of cellular biohybrids by employing cytoPNAs through reversible and tunable non-covalent interactions.^30,36^ ‘Cellnex’ technology presents a typical technology and has been extensively investigated for cellular biohybrid fabrication *via* polyphenol-based surface functionalization. Typically, polyphenol-functionalized nanocomplexes are first attached to the surface of targeted cells and provide multifunctional adsorption sites for further cytoPNAs to bind. The simplicity and modularity of ‘Cellnex’ enable the functionalization of red blood cells with a diverse array of biomolecules, encompassing functional proteins, DNA, mRNA, and viral vectors, all while preserving the intrinsic biological characteristics of the cells (Fig. 4A).^45^

The cytoPNA-mediated cell engineering performs as a less invasive strategy and the biohybrids can harness the functionals of both encapsulated cells and attached functional cargoes. For example, cytoPNA-assisted conjugation of PD-L1 monoclonal antibodies to the surfaces of macrophages facilitates the targeted accumulation of antibody-based therapeutics at tumor sites, thereby promoting immune response (Fig. 4B). The chemotactic mobility and effective tumor-homing capabilities of macrophages can be preserved in a cytoPNAs composed of polyphenol-functionalized polymeric gel on the surfaces of tumor-homing macrophages. Upon exposure to the acidic conditions characteristic of the tumor microenvironment, the disassembly of the metal–phenolic interactions facilitates the controlled release of therapeutics from the nanomatrix at the targeted site, realizing the precision of delivery (Fig. 4C).^46^ CytoPNAs have also been reported to control the cellular behavior of immune cells, such as maintaining the anti-inflammatory phenotype of regulatory T cells when attached on the surface, further guiding macrophage polarization, enhancing stromal cell osteogenic differentiation, and promoting hard tissue reparation.^47^

Other than therapeutic applications, cytoPNAs can enable convenient and rapid cell labeling *via* a non-transmembrane approach. This technique harnesses the adhesive properties of catechol groups to non-invasively coat the surfaces of cells with fluorescent markers, thereby offering a novel strategy for *in vivo* cell tracing and imaging.^48^ Leakage and loss prevention of the functional components in cells are significant for vaccine technologies. Intracellular materials from cancer cells can serve as novel cancer antigens, stimulating the immune system to develop a memory effect against such tumors, thereby inducing immune suppression. However, a significant challenge lies in minimizing the transmembrane diffusion of small molecules, such as RNA, into the extracellular environment. By constructing a manganese ion (Mn^2+^)-TA artificial cell membrane on the surface of cancer cells, we effectively reduce the diffusion of intracellular materials into the bloodstream, enabling precise delivery of immune cells and offering a new strategy for tumor immunotherapy (Fig. 4D).^49^

Although modifying the cell surface with cytoPNA can enrich cell function, ‘Cellnex’ has some challenges to consider in terms of cell surface functionalization. One of the primary challenges is the potential impact on cell viability and function. Although polyphenols provide a versatile platform for functionalizing cell surfaces, the encapsulation process can sometimes disrupt the cell membrane, leading to reduced cell viability or altered cellular behavior.^41,43^ Additionally, the stability of the polyphenol coatings under various physiological conditions needs further investigation. While these coatings are designed to be biocompatible, they may degrade or detach from the cell surface over time, affecting the longevity and effectiveness of the therapy.^42,45^ Furthermore, the complexity of the cellular microenvironment, including interactions with other cells and the extracellular matrix, can influence the performance of the encapsulated cells, making it essential to thoroughly understand and optimize these interactions for successful therapeutic outcomes.^47^ In general, the application of cytoPNAs in cell-based therapies offers a versatile and non-invasive platform for enhancing cellular function and therapeutic precision. The modularity and biocompatibility of phenolic chemistries hold significant promise for the next generation of cell therapies, from cancer treatment to vaccine development.

## 5 CytoPNAs on microbes for enhanced biomedical performance

Utilizing cytoPNAs for biohybrids has emerged as a versatile and powerful approach for developing advanced microbial-based therapies, where bacteria have drawn great attention while fungi, viruses, and algae are also employed. This strategy facilitates the targeted delivery, viability, and stability of microbia, and the customized/personalized therapeutic strategies, providing a robust foundation for applications in bioimaging, diagnostics, and treatments on diseases such as inflammatory bowel disease (IBD), cancer, and other immune-related disorders.

### 5.1 Microbial protection and enhanced targeted delivery

The efficacy of microbial-based therapies often faces significant obstacles such as low cell viability and off-target delivery, due to the encountered hostile environments of the gastrointestinal tract.^50^ CytoPNAs offer a robust solution to these challenges, either by encapsulating the bacterial cells for protection or utilizing the strong binding effect of catechol and galloyl groups with the endothelium cells in the gastrointestinal tract to assist bacterial colonization.

**Fig. 5:**
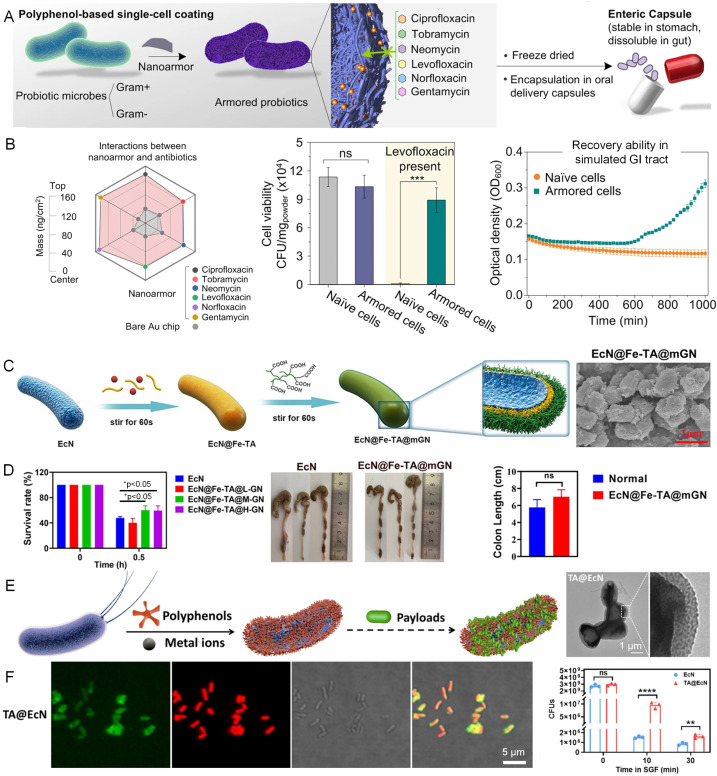
CytoPNAs on the cell surface to provide protection and enhance colonization of microbes in the gastrointestinal tract. (A) The polyphenol-based nanoarmor facilitates rapid, biocompatible bacterial cell surface functionalization, offering broad-spectrum protection against antibiotic treatments. (B) The interactions of polyphenol-based nanoarmor and various antibiotics. Polyphenol-based nanoarmor can improve the viability of bacteria in simulated gastrointestinal (GI) tract.^52^ Copyright 2022, Springer Nature. (C) Engineered bacterial cells by cytoPNAs *via* metal–phenolic coordination and synthetic polymers *via* layer-by-layer techniques. (D) Survival rate of bacteria with different loading amounts of functional cargoes on the cell surface, and the corresponding lengths of colons harvested from mice.^54^ Copyright 2023, American Chemical Society. (E) CytoPNAs induced bacterial cell surface functionalization with payloads. (F) Fluorescence characterization indicates the successful preparation of the nanocoatings on the bacterial cell surface. The survival rate of engineered bacteria being exposed to drugs.^55^ Copyright 2023, Elsevier. Statistical analysis was performed using one-way analysis of variance (ANOVA) analysis and Student's *t*-test. *P < 0.05, **P < 0.01, ***P < 0.001, and ****P < 0.0001 represented different statistical significances. ns stands for not significant.

Metal–phenolic nanocomplexes have shown effective in enhancing the viability and stability of anaerobic bacteria (*Bacteroides thetaiotaomicron*) by providing robust protection against oxygen exposure and processing stressors.^51^ TA and Fe^3+^ ions can assemble to form a nanoarmor on the bacterial cell surface, protecting the bacteria from the antibiotics in the gastrointestinal tract (Fig. 5A and B). This nanoarmor effectively shields both Gram-positive and Gram-negative bacteria from various antibiotics and promotes their colonization.^52^ In addition, combining the metal–phenolic coordination and layer-by-layer assembly with mucin and carboxymethylated β-glucan has significantly enhanced the colonization and targeted delivery of live bacterial therapeutics, particularly in the treatment of colitis (Fig. 5C and D).^53,54^ This versatility has also been extended to carrying diverse therapeutic cargoes, including small drug molecules and macromolecular proteins, enabling multimodal therapy across various biointerfaces such as the gastrointestinal tract, skin, and mucosae (Fig. 5E and F).^55^

Polyphenols have also been pivotal in directly forming nanocoatings that advance the viability and targeting capability of biohybrids. For instance, a double-layer coating of TA and enteric L100 has been employed to optimize the selective release and retention of the bacteria (*Escherichia coli* Nissle 1917) within the gastrointestinal tract, enhancing its therapeutic potential against conditions like colitis.^56^ Curcumin-loaded liposomes can also be attached to the microbial cell surface to further boost bacterial survival and adhesion.^57^ Moreover, hydrogel microspheres designed for targeted delivery provide a sophisticated platform for the co-delivery of probiotics and auxiliary molecules (indole-3-propionic acid, IPA), demonstrating their efficacy in modulating flora in the gastrointestinal tract and reducing intestinal inflammation.^58^ These strategies underscore the vital role of cytoPNAs in advancing microbial-based biohybrids, offering a refined approach for microbial protection and targeted delivery.

### 5.2 Tailored therapeutic actions

In microbial-based therapy, apart from focusing on microbial activity and targeted delivery, it is also significant to employ the appropriate therapeutic cargoes to construct cellular biohybrids for the correct therapeutic actions on specific diseases. Ideally, the microbial-based therapeutic biohybrids are expected to accurately reach the lesion and effectively intervene in the disease pathological process.

**Fig. 6:**
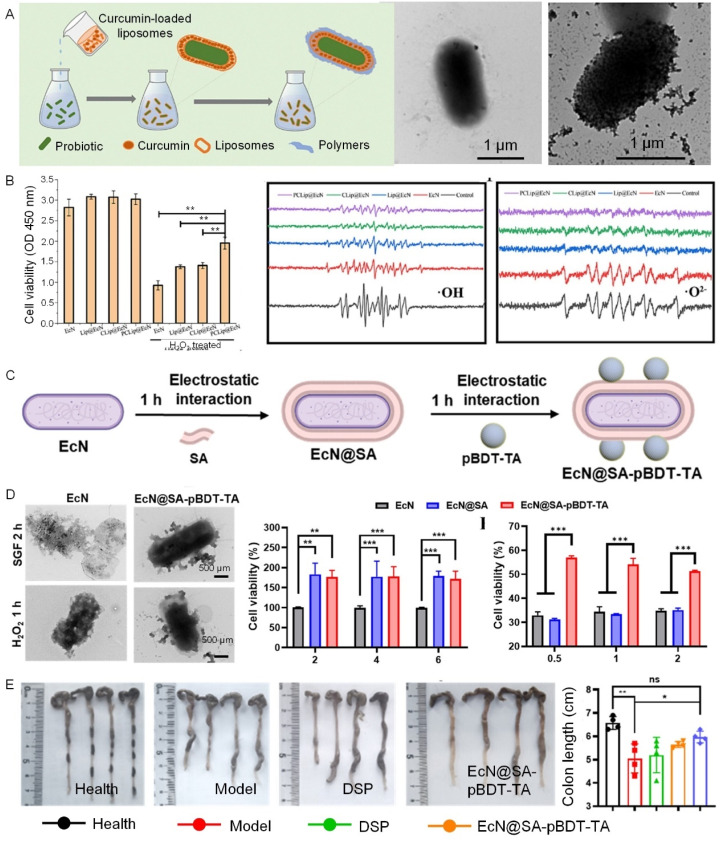
Tailored therapeutic actions enabled by bacterial cell surface functionalization with cytoPNAs. (A) Schematic illustration of the sequential process of preparation of bacterial-based biohybrids. (B) Cell viability under H_2_O_2_ treatment and scavenging effects of different treatments on hydroxyl (·OH) and superoxide (·O^2−^) radicals toward surface-modified bacteria.^57^ Copyright 2023, Elsevier. (C) Schematic illustration of the bacterial cell functionalization with a sequential process of polyphenol-based molecular interactions and layer-by-layer techniques. (D) The survival rate of bacteria under the treatment of active radical species. (E) Schematic diagram of colitis model mice and administration methods.^60^ Copyright 2024, American Chemical Society. Statistical analysis was performed using one-way analysis of variance (ANOVA) analysis and student's *t*-test. *P < 0.05, **P < 0.01, ***P < 0.001, and ****P < 0.0001 represented different statistical significances. ns stands for not significant.

Polyphenol-based surface functionalization offers a versatile platform for imparting bacterial biohybrids with a diverse range of therapeutic cargoes, including small molecule drugs, antibodies, enzymes, and gene therapies.^56,59^ By combining the inherent antioxidant and anti-inflammatory properties of polyphenols, these bacterial-based biohybrids can be precisely tuned to respond to specific stimuli in diseased tissues, enhancing the therapeutic potential (Fig. 6A and B).^57^ For instance, a nanostructured polyphenol-based platform (polymerizable aromatic dithiol-TA with sodium alginate) has been developed to enhance the bacteria resistance to oxidative and inflammatory stress, thereby improving the colonization and therapeutic efficacy in IBD treatment (Fig. 6C–E).^60^ This strategy allows the bacterial-based biohybrids with therapeutic actions of stimuli responsiveness for drug release at desired lesions.

Due to the edibility and biosafety, cytoPNA-containing biohybrids are primarily designed for gastrointestinal diseases, while preserving the potential for cancer treatment. The intratumoral microbiota plays a critical role in cancer progression and therapy, influencing tumor development and the immune response through mechanisms such as chronic inflammation and signaling pathway activation.^61^ Engineered bacteria, modified to deliver therapeutic agents directly to tumors, have shown promise in modulating the tumor immune microenvironment.^62,63^ Although cytoPNAs have yet to be widely applied in tumor therapy, the inherent properties of polyphenols, including their ability to inhibit tumor cell growth, prevent metastasis, and activate the immune response, indicate strong potential for future cancer treatments by tailoring therapeutic actions.^15,46,49,64^ Utilizing cytoPNAs to modify bacteria could enhance their ability to regulate tumor microenvironments, offering a novel approach to targeted cancer therapy by combining microbial colonization capabilities.

### 5.3 Bioimaging and diagnostics

CytoPNAs also hold wide potential applications in bioimaging and diagnostics. By integrating metal-based photosensitizers, magnetic nanoparticles, and carbon-based nanomaterials, polyphenol-based biohybrids can be engineered to enhance imaging capabilities.^65^ This integration allows for real-time visualization of bacterial biohybrids within the body, which is particularly valuable for tracking the efficacy of therapies, such as monitoring probiotic colonization in gastrointestinal diseases or visualizing tumor-targeting bacteria in cancer treatments. Additionally, the stability and biocompatibility of polyphenol-based coatings could enhance diagnostic accuracy and sensitivity, enabling earlier and more precise disease detection, as well as facilitating advancements in non-invasive monitoring and personalized medicine.

Although cytoPNAs of metal–phenolic nanocomplexes have been applied to a variety of bacterial species, expanding the range of applications in microbial protection and targeted delivery,^66^ several pitfalls are worth considering. The potential toxicity of the metal ions used in the cytoPNAs would be the first concern. While metal ions like Fe^3+^ and zinc ions (Zn^2+^) are generally biocompatible, other metal ions may exhibit cytotoxic effects, especially at high concentrations.^65^ Additionally, the choice of polyphenols must be tailored to the specific application, as different polyphenols can influence the stability and functionality of the prepared cytoPNAs.^52,60^ Furthermore, the stability of the coatings under physiological conditions and their impact on bacterial metabolic activity need to be thoroughly evaluated.^51,56^ Addressing these challenges will be essential for the successful development and clinical translation of cytoPNA–bacterial biohybrid for biomedical applications.

## 6 Conclusion and outlook

Natural polyphenols possess rich catechol or galloyl groups in the molecular structures, which can effectively impart novel functionalities or regulate surface properties of a wide range of substrates, including inorganic particles, organic polymers, and biomacromolecules. This polyphenol-mediated surface functionalization strategy has enabled a series of nanoarchitectures termed cytoPNAs, where the surface-exposed phenolic groups impart cell-adhesion properties. Both the polyphenols and cytoPNAs can be employed in cell surface functionalization in a cell-agnostic manner. The rapid formation and modular composition of cytoPNA-mediated cell engineering offer a powerful tool to overcome challenges encountered in biomanufacturing and cell-based therapies. In this Perspective, we present a discussion on the advances in cell surface modification using cytoPNAs. We discuss the molecular interactions that underpin the formation of cytoPNAs and their integration with engineered cells. The non-covalent interactions realize the capability and versatility of cytoPNA-mediated cell engineering in regulating cell survival and behaviors with new functionalities. The use of cytoPNAs to create inorganic–organic biohybrids can promote the production of high-value chemicals from cells, harnessing both the increased energy utilization and the regulation of the biosynthetic pathway. CytoPNAs can also advance cell- and microbial-based therapies with tailored therapeutic actions. We highlight the non-invasive surface functionalization for the enhancement of therapeutic precision, exhibiting the promise of cytoPNAs in therapies and vaccine development. In general, these versatile, dynamic, and biocompatible strategies open up new windows for next-generation cell engineering and beyond.

Despite the versatility and advantages of polyphenols and cytoPNAs in bio-manufacturing and cell-based therapy, they have limited applications due to ongoing challenges that need to be addressed before further exploration. From the technical perspective, preserving essential cell functions such as adhesion, proliferation, signaling, and immune interactions is crucial for cell surface modification. The non-covalent interactions among catechol or galloyl groups in cytoPNAs with cells are generally mild, reducing the risk of disrupting membrane proteins and receptors critical for cell functions. CytoPNAs of TA–Fe nanocomplexes attached to the bacterial cell surface are engineered sufficiently thin and porous, allowing for the free diffusion of small molecules and the unhindered operation of transmembrane receptors.^52,67^ The thickness and distribution densities of cytoPNAs on the cell surface are key parameters to ensure the surface modification does not impede the cellular process. In addition, the nanostructure of cytoPNAs also determines the success of cell surface modification, which can be turned *via* the parameters of compositions, molar ratios, pH, and ionic strength. CytoPNAs with high stability by fine-tuning the parameters play an essential role in mitigating the risk of vascular occlusion due to the potential aggregation. Ongoing research into the *in vivo* behavior of these nanocomplexes is crucial to ensure their safety in clinical applications. Moreover, the tunable nature of cytoPNAs allows for the incorporation of specific ligands or peptides that can facilitate or mimic natural adhesion processes, thereby supporting the formation of functional immunological synapses.^68^ Apart from the widely studied pH variations, additional mild and diverse stimuli such as temperature, enzyme activity, or specific molecular triggers can be integrated into the cytoPNAs to control cargo release.^69^ From the regulatory perspective, challenges lie in the urgent need for comprehensive validation of safety, efficacy, and reproducibility for clinical applications. The long-term biosafety profile and the *in vivo* metabolism of cytoPNAs are also critical concerns that needed for further assessments in clinical applications. Scalable and standardized fabrication processes for cytoPNAs are essential and require interdisciplinary collaboration.

Several directions can be proposed for further development of cytoPNA-mediated cell-engineering:

- Advanced theragnostic technology: rational design cytoPNAs with responsiveness to light, sound, and magnetic fields at physiological conditions to combine the inherent targeted delivery with photo- and sono-based therapy and diagnosis.

- Enhanced targeted delivery for plants: cytoPNAs for targeted drug delivery can be expanded from biomedicine to agriculture, which can promote the efficacy of pesticides and fertilizers to facilitate crop production and alleviate the current environmental pollution caused by indiscriminate usage.

- Artificial intelligence-assisted cell engineering platforms: metal–phenolic chemistry has plenty of combinations due to the variations of both polyphenols and metal ions. Machine learning algorithms can be trained to predict the properties of metal–phenolic complexes, accelerate the discovery process, and optimize drug release and cellular uptake profiles.

- Optimized fabrication for clinical translation: polyphenol is generally considered safe as a food additive and adjuvant in pharmaceutics. Certain metal ions and most biomacromolecules are also clinically approved. Clinical translation of cytoPNAs would accelerate the development of novel therapies. Chemically demonstration of the uniformity of the assemblies in composition, size, and morphology from different batches would be the primary goal to reach.

## Data availability

No primary research results, software or code have been included and no new data were generated or analysed as part of this review.

## Author contributions

Conceptualization: J. Guo and Y. He; writing – original draft: Y. He; writing – review & editing: Y. He, Q. Liu, Y. He and S. Deng; validation: Y. He and J. Guo.

## Conflicts of interest

The authors have no conflicts to disclose.
